# Locally Acquired (Autochthonous) Mosquito-Transmitted *Plasmodium vivax* Malaria — Saline County, Arkansas, September 2023

**DOI:** 10.15585/mmwr.mm7342a2

**Published:** 2024-10-24

**Authors:** Ashleah P. Courtney, Bobby L. Boyanton, Paige V. Strebeck, Keith Blount, Savanna Ledford, Alison D. Ridpath, Kimberly E. Mace, Cherie Smith, Kelley Garner, Catherine Waters, Michael J. Cima, Naveen Patil, Peter D. McElroy, Brian H. Raphael, Sarah G.H. Sapp, Yvonne Qvarnstrom, Audrey Lenhart, Alice Sutcliffe, Arkansas Locally Acquired Mosquito-Transmitted Malaria Response Team, Theresa M. Dulski, Laura Rothfeldt, Arkansas Locally Acquired Mosquito-Transmitted Malaria Response Team, Danny Baxter, Monica Baxter, Lisa Collier, Claudia Corredor-Medina, Melissa Green, Stephen Hedges, Leslie Himstedt, Katelyn Lazenby, Laura Leite, Cole Menasco, Meg Mirivel, Emma Rogers, Jennifer Shray, Leah Welker

**Affiliations:** ^1^University of Arkansas for Medical Sciences, Little Rock, Arkansas; ^2^Arkansas Children’s Hospital, Little Rock, Arkansas; ^3^Arkansas Department of Health; ^4^Epidemic Intelligence Service, CDC; ^5^Division of Parasitic Diseases and Malaria, National Center for Emerging and Zoonotic Infectious Diseases, CDC; ^6^Career Epidemiology Field Officer Program, CDC.; Arkansas Department of Health; Arkansas Department of Health; Arkansas Department of Health; CDC; Arkansas Department of Health; Arkansas Department of Health; Arkansas Department of Health; Arkansas Department of Health; CDC; Arkansas Department of Health; Arkansas Department of Health; CDC; Arkansas Department of Health; Arkansas Department of Health.

**Summary**

**What is already known about this topic?**

After 20 years without locally acquired mosquito-transmitted malaria in the United States, nine cases were reported to CDC during May–August 2023.

**What is added by this report?**

In September 2023, a 10th U.S. case of locally acquired malaria was diagnosed, in Arkansas. The public health response included case investigation and surveillance, mosquito surveillance and control, assessment of hospital preparedness, and clinical and public outreach.

**What are the implications for public health practice?**

Prompt diagnosis and appropriate treatment of malaria can improve clinical outcomes and lower risk for ongoing transmission. Although the risk for locally acquired malaria in the United States remains very low, its reemergence highlights the importance of vectorborne disease preparedness and response efforts.

## Abstract

A case of locally acquired (autochthonous) mosquito-transmitted *Plasmodium vivax* malaria was diagnosed in Arkansas in September 2023. This represents the 10th autochthonous case identified nationally in 2023, after 20 years without recorded local mosquitoborne malaria transmission in the United States. The public health response included case investigation, active case surveillance, mosquito surveillance and control, assessment of medical countermeasures, and clinical and public outreach. Prompt diagnosis and appropriate treatment of malaria can improve clinical outcomes and, in addition to vector control, minimize risk for local transmission. Clinicians should consider malaria among patients who have traveled to countries where malaria is endemic, or with unexplained fever regardless of travel history. Although the risk for autochthonous malaria in the United States remains very low, its reemergence highlights the importance of vectorborne disease preparedness and response. Examples of such efforts include improving awareness among clinicians, access to diagnostics and antimalarial medications, and capacity for mosquito surveillance and control. Collaboration and communication among CDC, health departments, local jurisdictions, clinicians, hospitals, laboratories, and the public can support rapid malaria diagnosis, prevention, and control. Before traveling internationally to areas where malaria is endemic, travelers should consult with their health care provider regarding recommended malaria prevention measures, including chemoprophylaxis and precautions to avoid mosquito bites, to reduce both personal and community risk.

## Investigation and Results

On September 28, 2023, a previously healthy person living in Saline County, Arkansas was evaluated at a local hospital for a 10-day history of headache, fever, chills, night sweats, fatigue, and a 1-day history of nausea and vomiting. The patient had no reported history of international travel, blood transfusion, organ transplant, or other bloodborne pathogen exposure. Initial laboratory evaluation revealed anemia, thrombocytopenia, and hyperbilirubinemia. The patient was admitted for possible hematologic malignancy; anemia and thrombocytopenia worsened (hemoglobin 7.3 g/dL [reference range 12.0–16.0 g/dL]; platelets 14 K/µL [reference range 150–400 K/µL]), and the patient was transfused one unit of packed red blood cells and one unit of platelets.

On repeat complete blood count analysis (September 30, 2023), the patient’s thin peripheral blood smear was noted to have ring forms concerning for malaria and verified 7 hours later as *Plasmodium* (non-*falciparum*) by positive result from a BinaxNOW malaria rapid diagnostic test (RDT; Abbott). *Plasmodium* species and parasitemia percentage were initially unavailable; the patient was treated with intravenous (IV) artesunate pending results.^†^ A pathologist’s review of thick and thin blood smears verified presence of *Plasmodium vivax/Plasmodium ovale* gametocytes and ring forms with parasitemia of 0.26%. The patient was transitioned to artemether-lumefantrine and primaquine for relapse prevention. Thin smear images were referred to CDC for telediagnosis, which confirmed the presence of *P. vivax/P. ovale*. CDC performed direct morphologic examination of thin smears (Figure) followed by 18S rRNA real-time polymerase chain reaction testing of ethylenediaminetetraacetic acid whole blood, which confirmed the species-level identity of *P. vivax.* To exclude transfusion-associated malaria, a pretransfusion peripheral blood smear was re-reviewed by both pathologist-in-chief at the local hospital and CDC and confirmed to have ring forms and gametocytes consistent with *P. vivax/P. ovale*. The patient completed treatment and fully recovered.

**FIGURE:**
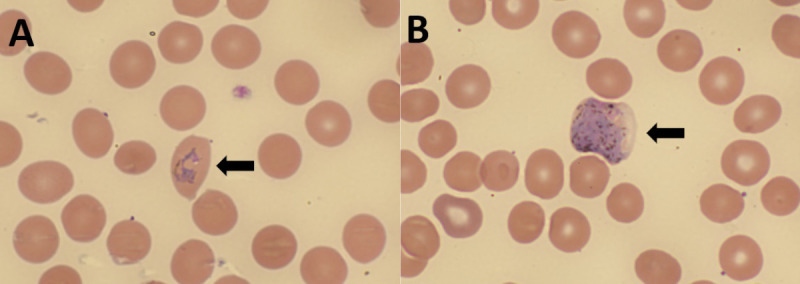
Thin blood smear* from the patient, demonstrating *Plasmodium vivax/ovale* ring-form trophozoite (A) and gametocyte (B) — Arkansas, September 2023 Photos/Arkansas Children’s Hospital, Clinical Microbiology Laboratory * Wright-Giemsa stain (x1000 magnification).

## Public Health Response

CDC supported the Arkansas Department of Health (ADH) in the investigation and response to this case. This activity was reviewed by CDC, deemed not research, and was conducted consistent with applicable federal law and CDC policy.^§^

### Additional Case Investigation

ADH confirmed the patient had never traveled internationally and did not have other risk factors for acquiring malaria in the United States. Outdoor activities and possible mosquito exposure locations during the 4 weeks before symptom onset were assessed; these did not overlap geographically or temporally with any 2023 imported malaria cases in Arkansas, which were all caused by *P. falciparum.*

### Active Case Surveillance

ADH confirmed that all household members living with the patient were asymptomatic. On October 9, 2023, prospective enhanced case finding was implemented using the Electronic Surveillance System for the Early Notification of Community-Based Epidemics (ESSENCE) syndromic surveillance system. Of eight local hospitals in Saline County and Little Rock, three were operational within ESSENCE, two were onboarded but not yet fully operational, and three were not participating. The ADH syndromic surveillance team adapted a query originally developed by the Florida Department of Health to identify potential malaria cases^¶^ (*1*). Syndromic surveillance continued for 9 weeks after the patient’s symptom onset. Eight potential malaria cases were identified; all patients received alternative diagnoses.

### Mosquito Surveillance and Control

On October 5, 2023, ADH began enhanced mosquito surveillance at two sites in Saline County near the patient’s residence and a location where the patient spent time outdoors during the preceding 4 weeks. Five CDC miniature light traps with and without carbon dioxide, which is a mosquito attractant, were set up at each site during the early evening and collected approximately 14 hours later the next day, October 6, 2023. This process was repeated six additional times, with the final mosquito collection occurring on October 24, 2023. Of the 244 total mosquitos collected, 25 female *Anopheles* mosquitoes were identified and sent to CDC for *Plasmodium* testing**; all specimens tested negative.

ADH provided guidance on mosquito control efforts based on local capabilities in consultation with CDC.^††^ The affected municipality lacked routine mosquito control capabilities and partnered with another municipality that donated adulticide, an ultra-low volume (ULV) sprayer, and a dedicated spray truck. On October 5, 2023, the affected municipality started control efforts for adult mosquitoes near the patient’s residence. The ULV truck-mounted sprayer treated for 5 nonconsecutive nights over a 7-day period using 30% permethrin (active ingredient)/30% piperonyl butoxide (synergist). Control efforts started within 1 mile (1.6 km) of the patient’s residence, expanding for two additional nights in 1-mile (1.6-km) concentric rings, where roads permitted travel. The final two nights focused on potential mosquito breeding areas near the patient’s residence and another location where the patient spent time outdoors. Control efforts were discontinued after treatment on October 11, 2023, because of resource limitations and falling temperatures in the area, which can reduce mosquito activity.

### Assessment of Medical Countermeasures

Starting October 6, 2023, local hospitals in Saline County and Little Rock were contacted to assess malaria diagnostic and treatment capabilities. Seven of eight local hospitals reported ability to examine and interpret thick and thin blood smears, and two reported use of malaria RDTs. The Arkansas State Public Health Laboratory can consult on blood smears upon request and refer slides and whole blood as needed to CDC.^§§^ Three hospitals reported availability of artemether-lumefantrine, the first-line drug in the United States for uncomplicated *P. falciparum* or unknown malaria species. No hospitals stocked IV artesunate, the first-line drug for treatment of severe malaria in the United States.^¶¶^

### Clinical and Public Outreach

On October 4, 2023, ADH issued a press release notifying the public of a case of locally acquired mosquito-transmitted malaria. The press release included information about malaria symptoms, importance of seeking medical care if symptomatic, and mosquito control and bite prevention.***

On October 5, 2023, ADH distributed a Health Advisory (Identification of Locally Acquired Mosquito-Transmitted Malaria in Arkansas) via email through the Arkansas statewide Health Alert Network. This advisory provided guidance to clinicians, hospitals and laboratories and included recommendations and resources related to malaria clinical presentation, diagnosis, treatment, prevention, and case reporting. On October 11, 2023, ADH hosted a 1-hour informational webinar for clinicians to discuss the health advisory and highlight available resources.

## Discussion

The total number of malaria cases reported in the United States trended upward during 1972–2019, with 2,048 cases reported to CDC in 2019 (*2*). Most of these cases were associated with travel to 85 countries where malaria remains endemic and could represent a potential source of *Plasmodium* infection for locally acquired mosquito-transmitted cases (*3*). *Anopheles* mosquito species are present across the United States and can acquire *Plasmodium* infection from patients with travel-associated malaria; these competent vectors can then transmit the parasite to persons who haven’t traveled (*4*). Although the source of this autochthonous malaria case in Arkansas remains unknown, local *Anopheles* mosquitoes might have become infective after obtaining a blood meal from a person with undiagnosed travel-associated malaria in a nearby geographic area (*4*). This represents the 10th autochthonous malaria case reported to CDC in 2023 after 20 years without recorded local transmission, thus highlighting the ongoing need for coordinated public health response and prevention efforts (*4*,*5*).

Prompt diagnosis and appropriate treatment^†††^ of malaria can improve clinical outcomes and lower risk for ongoing local transmission (*5*). Although the risk for locally acquired mosquito-transmitted malaria in the United States remains very low, clinicians should consider malaria for all patients who have traveled to countries where malaria is endemic, or who have unexplained fever, regardless of travel history (*5*). The Clinical and Laboratory Standards Institute (CLSI) developed guidelines to support accurate and timely malaria diagnosis, including recommendations for specimen collection, blood film preparation, staining procedures, and identification of *Plasmodium* spp.^§§§^ Many U.S. laboratories report the capability to perform the gold-standard malaria diagnostic test: microscopic examination of thick and thin blood smears; however, few adhere to all CLSI guidelines, which could contribute to diagnostic delays (*6*). Resources such as proficiency testing programs, guidelines, bench aids, continuing education workshops, and telediagnosis are available for maintenance and improvement of laboratory capacity (*6*–*8*). RDTs can be used to aid diagnosis but should be used alongside microscopy because they are less sensitive and cannot reliably confirm species-level identification or determine parasitemia density (*5*). Stocking IV artesunate, the first-line drug for treatment of severe malaria in the United States, might improve timeliness of patient treatment and minimize risk for death; emergency procurement is also available (*9*). Treatment of uncomplicated malaria varies by *Plasmodium* species, expected drug susceptibility, and previous use of antimalarials (*9*). If primaquine or tafenoquine are indicated, quantitative glucose-6-phosphate dehydrogenase (G6PD) testing should be conducted before administration because of risk of hemolytic anemia and need for a modified regimen or alternative medication if G6PD deficiency is detected (*9*).

Malaria is a nationally notifiable condition, and case reporting guides local and national prevention and response activities (*10*). Enhanced case finding might also occur through syndromic surveillance, highlighting the importance of participation of local health care facilities in state syndromic surveillance systems. Vectorborne disease outbreak preparedness varies by jurisdiction; local partnerships and resource sharing can improve vector control capacity (*4*). Collaboration and communication with clinicians, hospitals, laboratories, and the public can support rapid malaria identification, prevention, and control. As of September 2024, no additional autochthonous malaria cases had been identified in Arkansas. Before traveling internationally to areas where malaria is endemic, travelers should consult with their health care provider regarding recommended malaria prevention measures, including chemoprophylaxis and precautions to avoid mosquito bites, to reduce both personal and community risk.

## Footnotes

* These senior authors contributed equally to this report.

† IV artesunate is the first-line drug for treatment of severe malaria in the United States. Clinical manifestations of severe malaria include impaired consciousness, seizures, circulatory collapse/shock, pulmonary edema or acute respiratory distress syndrome, acidosis, acute kidney injury, abnormal bleeding or disseminated intravascular coagulation, severe anemia (hemoglobin <7 g/dL), and high percent parasitemia (≥5%). https://www.cdc.gov/malaria/hcp/clinical-guidance/treatment-of-severe-malaria.html

§ 45 C.F.R. part 46, 21 C.F.R. part 56; 42 U.S.C. Sect. 241(d); 5 U.S.C. Sect. 552a; 44 U.S.C. Sect. 3501 et seq.

¶ The query used *International Classification of Diseases, Tenth Revision* codes and free text to identify references to 1) malaria or *Plasmodium* spp., 2) fever or chills, abdominal pain, and anemia or thrombocytopenia (with terms to exclude records without fever), or 3) splenomegaly. The ESSENCE system algorithm identified records matching query parameters and alerted epidemiologists by email. Raw data in the Rhapsody Integrated Development Environment were monitored twice daily through a parallel Structured Query Language data query used to search Health Level 7 messages from the facilities in staging.

** Mosquitos were tested by both 1) bead assay for circumsporozoite protein in head-thorax for *P. falciparum, P. vivax* vk210 and *P. vivax* vk247, and 2) *Plasmodium* cytochrome c oxidase subunit I polymerase chain reaction using DNA extracted from abdomens.

†† CDC provides clinical assistance through the Malaria Hotline at 770-488-7788 or 855-856-4713 (toll-free) Monday–Friday, 9:00 a.m.–5:00 p.m. ET. After hours, on weekends, and on federal holidays, health care providers can call 770-488-7100 and ask to speak with the malaria clinician on call and diagnostic laboratory support. https://www.cdc.gov/parasites/contact/?CDC_AAref_Val=https://www.cdc.gov/parasites/contact.html

§§ ADH ordered 15 boxes of malaria RDT kits to help support local malaria diagnostics.

¶¶
https://www.cdc.gov/malaria/hcp/clinical-guidance/iv-artesunate-us.html?CDC_AAref_Val=https://www.cdc.gov/malaria/diagnosis_treatment/artesunate.html

***
https://healthy.arkansas.gov/article/locally-acquired-malaria-identified-in-arkansas/

†††
https://www.cdc.gov/malaria/hcp/clinical-guidance/index.html

§§§
https://clsi.org/standards/products/microbiology/documents/m15/
